# Rapid and high throughput molecular identification of diverse mosquito species by high resolution melting analysis

**DOI:** 10.12688/f1000research.9224.1

**Published:** 2016-08-11

**Authors:** Yvonne Ukamaka Ajamma, Enock Mararo, David Omondi, Thomas Onchuru, Anne W. T. Muigai, Daniel Masiga, Jandouwe Villinger

**Affiliations:** 1 Martin Lüscher Emerging Infectious Diseases (ML-EID) Laboratory, International Centre of Insect Physiology and Ecology, Nairobi, Kenya; 2 Department of Botany (Genetics), Jomo Kenyatta University of Agriculture and Technology, Juja, Kenya; 3 Biochemistry and Molecular Biology Department, Egerton University, Egerton, Kenya; 4 Molecular Biology and Virology Laboratory, Department of Medical Biosciences, University of Western Cape, South Africa; 5 Insect Symbiosis Research Group, Max Planck Institute for Chemical Ecology (MPI-CE), Jena, Germany; 6 Department for Evolutionary Ecology, Institute for Zoology, Johannes Gutenberg University, Mainz, Germany

**Keywords:** High resolution melting analysis, molecular identification, mosquitoes, Aedes, Culex, Mansonia, Anopheles

## Abstract

Mosquitoes are a diverse group of invertebrates, with members that are among the most important vectors of diseases. The correct identification of mosquitoes is paramount to the control of the diseases that they transmit. However, morphological techniques depend on the quality of the specimen and often unavailable taxonomic expertise, which may still not be able to distinguish mosquitoes among species complexes (sibling and cryptic species). High resolution melting (HRM) analyses, a closed-tube, post-polymerase chain reaction (PCR) method used to identify variations in nucleic acid sequences, has been used to differentiate species within the *Anopheles gambiae *and *Culex pipiens *complexes. We validated the use of PCR-HRM analyses to differentiate species within *Anopheles* and within each of six genera of culicine mosquitoes, comparing primers targeting cytochrome b ( *cyt b*), NADH dehydrogenase subunit 1 (ND1), intergenic spacer region (IGS) and cytochrome c oxidase subunit 1 ( *COI*) gene regions. HRM analyses of amplicons from all the six primer pairs successfully differentiated two or more mosquito species within one or more genera ( *Aedes* ( *Ae. vittatus* from *Ae. metallicus*), *Culex *( *Cx. tenagius* from *Cx. antennatus*, *Cx. neavei* from *Cx. duttoni*, cryptic *Cx. pipiens* species), *Anopheles *( *An. gambiae s.s.* from *An. arabiensis*) and *Mansonia *( *Ma. africana* from *Ma. uniformis*)) based on their HRM profiles. However, PCR-HRM could not distinguish between species within *Aedeomyia *( *Ad. africana *and *Ad. furfurea*), *Mimomyia *( *Mi. hispida *and *Mi. splendens*) and *Coquillettidia *( *Cq. aurites*, *Cq. chrysosoma*, *Cq. fuscopennata*, *Cq. metallica*, *Cq. microannulatus*, *Cq. pseudoconopas* and *Cq. versicolor*) genera using any of the primers. The IGS and COI barcode region primers gave the best and most definitive separation of mosquito species among anopheline and culicine mosquito genera, respectively, while the other markers may serve to confirm identifications of closely related sub-species. This approach can be employed for rapid identification of mosquitoes.

## Introduction

Mosquitoes are among the most important disease vectors, known to transmit and maintain the circulation of pathogens that cause both global and neglected tropical diseases in humans and animals ^1^. The correct identification of different field-collected mosquito species, endemic to distinct ecologies, with high parasite and arthropod-borne virus (arbovirus) diversities is crucial to the planning of targeted vector control strategies to mitigate disease transmission ^2^. The last and most comprehensive Afrotropical mosquito identification keys were published in 1941 for culicines ^3^ and in 1987 for anophelines ^4^. Molecular approaches that efficiently differentiate conspecific mosquitoes such as the barcode region ^5^ improve identification accuracy considerably ^6^, but are time consuming, expensive in terms of post-polymerase chain reaction (post-PCR) processing and depend heavily on DNA sequencing.

Recent approaches have taken advantage of the unique melting profiles generated by homologous PCR products with small sequence differences during high resolution melting (HRM) analysis ^7, 8^. Indeed, PCR-HRM has been used to differentiate mosquito transmitted arboviruses ^9– 11^ and malaria *Plasmodium* ^12, 13^, vertebrate blood meals of mosquitoes ^10^, between two members of the *Anopheles gambiae* complex ^14^ and amongst three members of the *Culex pipiens* complex ^15^. HRM analysis has proven to offer higher resolution of PCR product based species identification on sequence variants than electrophoretic methods by revealing even single nucleotide polymorphisms (SNPs) in the simple sequence repeats (SSRs) among products of similar sizes ^16, 17^. Conventional detection of specific PCR products sequence relies on costly molecular probes and/or product sequencing ^18^. For species identification ^16^, only representative samples with distinct HRM profiles need to be sequenced, thereby reducing reagent and sample consumption costs ^10– 11^. Combining HRM analysis of barcode region sequences (Bar-HRM) has been successfully used to rapidly and accurately distinguish between closely related antelope species ^19^ and medicinal plants ^20, 21^ and to authenticate the source of vegetable oils ^22^.

Although HRM has been successfully used to differentiate between specific *Anopheles* and *Culex* mosquitoes, the approach’s broader applicability and most suitable markers have not been evaluated. Previously, only the ribosomal DNA was targeted for *An. gambiae sensu lato* ( *s.l.*) ^14^ and only the acetylcholinesterase gene was used in distinguishing the *Cx. pipiens* complex ^15^. This study aimed at validating the use of HRM analysis for high throughput molecular culicine and anopheline mosquito identification and differentiation, comparing the utility of one ribosomal IGS (previously used to differentiate *An. gambiae s.l.*) ^14^ and three mitochondrial (COI, ND1, *cyt b*) gene markers.

## Methods

### Sample collection and identification

We used 109 mosquitoes ( Table 1 and Table 2) that were collected in 2012 during the rainy seasons near Lake Baringo from March 2–4, July 16–24 and October 12–21 and Lake Victoria from April 2–15, May 18–31 and November 12–29 during a mosquito diversity study around the islands and mainland shores of Lake Baringo in Baringo County ( Table 1) and Lake Victoria in Homa Bay County ( Table 2) in Kenya ^6^. Before sampling, we obtained ethical clearance for the study from the Kenya Medical Research Institute (KEMRI) ethics review committee (Approval Ref: Non-SSC Protocol #310). These mosquitoes were morphologically identified during a previous study ^6^. Baringo County is a known hotspot for arbovirus outbreaks ^23^, while Homa Bay County is endemic to malaria and is located in a region with a history of arbovirus activity ^10^. One sample each of *Anopheles gambiae sensu stricto* ( *s.s.*) and *An. arabiensis, Aedes aegypti* and *Culex pipiens* from laboratory colonies maintained in the Insectary of the International Centre of Insect Physiology and Ecology ( *icipe*), Nairobi, Kenya, were used as controls. Also, specimens with confirmed identity that have been previously sequenced and submitted to GenBank ( Table 1 and Table 2) were used as both controls and samples.

**Table 1.** Number (N) of mosquito species (GenBank accessions) used for HRM analyses from Baringo County, Kenya.

Mosquito species	N	Logumgum 0.455 N, 36.078 E	Sirata 0.462 N, 36.097 E	Kampi ya Samaki 0.620 N, 36.028 E	Nosuguro 0.605 N, 36.126 E
*Ad. africana*	**4**	4 (KU186980, KU186981, KU186982, KU186985)			
*Ad. furfurea*	**4**		4 (KU186979, KU186983, KU186984, KU186986)		
*An. funestus*	**3**		3 (KU187102, KU187103, KU187105)		
*An. gambiae s.l.*	**3**		1	2	
*Cq. aurites*	**2**		2 (KU187114, KU187117)		
*Cq. chrysosoma*	**1**	1 (KU187115)			
*Cq. fuscopennata*	**1**				1 (KU187116)
*Cq. metallica*	**2**		2 (KU187112, KU187113)		
*Cx. antennatus*	**2**	1	1 (KU187050)		
*Cx. perexiguus*	**4**			4 (KU380423, KU380348, KU380476, KU380382)	
*Cx. tenagius*	**1**		1 (KU187054)		
*Ma. africana*	4		2 (KU187124, KU187130)		2 (KU187127, KU187128)
*Ma. uniformis*	3	2 (KU187170, KU187171)			1 (KU187164)
**Total**	**34**	**8**	**16**	**6**	**4**

GenBank accessions are provided only for samples with confirmed identity and from which the *COI* DNA sequences were obtained during a previously published mosquito diversity study ^6^.

**Table 2.** Number (N) of mosquito species (GenBank accessions) used for HRM analyses from Homa Bay County, Kenya.

Mosquito species	N	Mbita 0.432 S, 34.210 E	Luanda Nyamasare 0.478 S, 34.287 E	Ngodhe 0.505 S, 34.363 E	Ungoye 0.612 S, 34.098 E	Mfangano Island 0.462 S, 33.999 E	Rusinga Island 0.399 S, 34.193 E	Chamaunga Island 0.431 S, 34.228 E	Takawiri Island 0.472 S, 34.091 E
*Ae. metallicus*	**3**	1 (KU187014)			1 (KU187013)	1			
*Ae. vittatus*	**8**					6 (KU187004, KU187006, KU187008-KU187011)			2 (KU187005, KU187007)
*An. coustani s.l.*	**10**		3 (KU187098, KU187100, KU187101)	2 (KU187095, KU187096)			2 (KU187097, KU187099)	2	1
*An. funestus*	**2**	1 (KU187104)						1	
*An. gambiae s.l.*	**2**					2 (KU187108, KU187109)			
*Cq. aurites*	**1**	1 (KU187121)							
*Cq. microannulatus*	**2**					1		1 (KU187118)	
*Cq. pseudoconopas*	**1**		1						
*Cq. versicolor*	**2**			2 (KU187119, KU187120)					
*Cx. antennatus*	**4**	4 (KU187037, KU187038, KU187048)							
*Cx. duttoni*	**6**	1 (KU187075)				5 (KU187067, KU187068, KU187070-KU187072)	1		
*Cx. neavei*	**3**					3 (KU187032, KU187040, KU187046)			
*Cx. perexiguus*	**1**					1 (KU380445)			
*Cx. pipiens*	**6**					1 (KU187083)	5 (KU187077, KU380366, KU380372, KU380431, KU380444)		
*Culex* sp. GPA	**5**	3 (KU380352, KU380455, KU380394)					2 (KU380377, KU380413)		
*Cx. univittatus*	**3**	3 (KU187056, KU187059, KU187060)							
*Cx. watti*	**5**	2 (KU187063, KU187064)				2	1		
*Ma. africana*	**2**			1				1 (KU187153)	
*Ma. uniformis*	**3**		1 (KU380460)				1	1 (KU187175)	
*Mi. hispida*	**2**	2							
*Mi. splendens*	**3**	3 (KU187093, KU187094)							
**Total**	**75**	**21**	**5**	**5**	**1**	**22**	**12**	**6**	**3**

GenBank accessions are provided only for samples with confirmed identity and from which the *COI* DNA sequences were obtained during a previously published mosquito diversity study ^6^.

### DNA extraction

From each mosquito, we extracted DNA according to the hot sodium hydroxide and Tris (HotSHOT) DNA extraction protocol ^24^ from a single mosquito leg that was detached from the rest of the body using a pair of forceps and dissecting pin. Without crushing, the mosquito leg was put in a 0.2 ml microcentrifuge tube containing 30 µl of Alkaline Lysis buffer (25 mM NaOH (Thermo Fisher Scientific, Pittsburgh, USA), 0.2 mM disodium EDTA (Thermo Fisher Scientific), pH 8.0) and incubated in a thermocycler at 95°C for 30 minutes and cooled at 4°C for 5 minutes. Then, 30 µl neutralising solution (40 mM Tris-HCl (Thermo Fisher Scientific)) was added. The resulting DNA was stored at -20°C until required as templates for PCR assays.

### Primer design, PCR and HRM analyses

Based on multiple alignments using Geneious software version 8.1.4 ^25^ of mitochondrial genomes of mosquitoes (GenBank accessions NC_015079, NC_028616, NC_028223, KR068634, NC_010241, NC_014574, EU352212, NC_008070, KT358413, KT382816, KU494979, JX040513, AY729979, KU494979), we designed four sets of primers from two mitochondrial gene regions: *COI* (COI-AnophF/HCO2108R; Uni-Minibar-JVF/Uni-Minibar-JVR; Mos-CO1-JVF/Mos-CO1-JVR) and ND1 (Mos-ND1F/Mos-ND1R) genes ( Table 3). The COI AnophF primer was initially designed specifically for *Anopheles* mosquitoes to be used with the HCO2108R primer ^26^, but tested on other species as well. Using samples of morphologically and molecularly identified *Culex*, *Aedeomyia*, *Mimomyia*, *Coquillettidia*, *Mansonia*, *Aedes*, and *Anopheles* mosquito species ( Table 1 and Table 2), we amplified different gene regions of their genomes using six pairs of primers ( Table 3) in three replicate runs of single-plex PCRs in a Rotor-Gene Q HRM real time PCR thermocycler (QIAGEN, Hannover, Germany). PCR grade water was used as negative control while mosquito species from *Ae. aegypti*, *An. gambiae s.s.*, *An. arabiensis* and *Cx. Pipiens quinquefasciatus* colonies maintained in the International Centre of Insect Physiology and Ecology ( *icipe*) Insectary Unit were used as positive controls. The PCR mix contained 5 µl of 5X Hot Firepol EvaGreen HRM Mix (Solis BioDyne, Tartu, Estonia), 0.5 µM of each primer, 1 µl of DNA template and distilled water in a final volume of 10 µl. The thermal cycling conditions involved an initial denaturation for 1 minute at 95°C, followed by 35 cycles of denaturation at 95°C for 30 seconds, annealing at 50°C for 20 seconds, and extension at 72°C for 30 seconds, and a final extension at 72°C for 7 minutes. Without stopping the reaction, the PCR amplicons were denatured at 95°C for 1 minute, held for another minute at 40°C and melted by gradually raising the temperature from 70°C to 95°C by 0.1°C in 2 second steps, waiting for 90 seconds of pre-melt conditioning on first step and 2 seconds in subsequent steps. The outcome was automatically plotted on a connected computer and visually observed and analysed using the Rotor-Gene Q Series software v2.1. Representative samples of differentiated mosquito species that had similar HRM curves were purified with ExoSAP-IT (USB Corporation, Cleveland, OH) and submitted for DNA sequencing at Macrogen (South Korea). To confirm the identity of PCR-HRM differentiated mosquitoes, DNA sequences were edited with Geneious version 8.1.4 ^25^ and queried against the GenBank nr database ( http://www.ncbi.nlm.nih.gov/) using the Basic Local Alignment Search Tool (BLAST N) version 2.3.0 ^27^.

**Table 3.** Primers used for the amplification of gene fragments.

Target gene	Primer name	Primer Sequence (5’ to 3’)	Reference genome	Primer coordinates	Amplicon size (bp)
Mitochondrial COI (within barcode region)	COI-AnophF	GCAGGAATTTCTTCTATTTTAGG	L20934	1,874–1,896	275
	HCO2198R ^26^	TAAACTTCAGGGTGACCAAAAAATCA	L20934	2,148–2,123	
Mitochondrial COI	Uni-Minibar-JVF	ACAAATCATAARGATATTGGAAC	L20934	1,445–1,467	173
	Uni-Minibar-JVR	AAAATTATAATAAAWGCATGAGC	L20934	1,617–1,55	
Mitochondrial COI	Mos-Co1-JVF	ATAGTWATACCTATYATAATTGG	L20934	1,622–1,644	299
	Mos-Co1-JVR	ACWGTAGTAATAAAATTTACTGC	L20934	1,920–1,898	
Mitochondrial ND1	Mos-ND1F	TATGTCTTGAAAACATAAGAAAG	L20934	11,569–11,591	173
	Mos-ND1R	CGDTATGATAAATTAATGTAATTAG	L20934	11,717–11,741	
Mitochondrial *cyt b*	CYT BF ^35^	GGACAAATATCATTTTGAGGAGCAACAG	L20934	10,821–10,848	470
	CYT BR ^35^	ATTACTCCTCCTAGCTTATTAGGAATTG	L20934	11,290–11,263	
Ribosomal DNA IGS	AgamUni F ^2^	GTGAAGCTTGGTGCGTGCT	KT284724	126–174	169
	AgamUni R ^2^	GCACGCCGACAAGCTCA	KT284724	319–303	

F is forward primer direction; R is reverse primer direction.

## Results

Raw Rotor-Gene Q HRM data files (.rex), viewable using Rotor-Gene Q software (Qiagen) ‘Contents.csv’ contains a description of the files. Click here for additional data file. Copyright: © 2016 Ajamma YU et al. 2016 Data associated with the article are available under the terms of the Creative Commons Zero "No rights reserved" data waiver (CC0 1.0 Public domain dedication).

**Figure 1.**
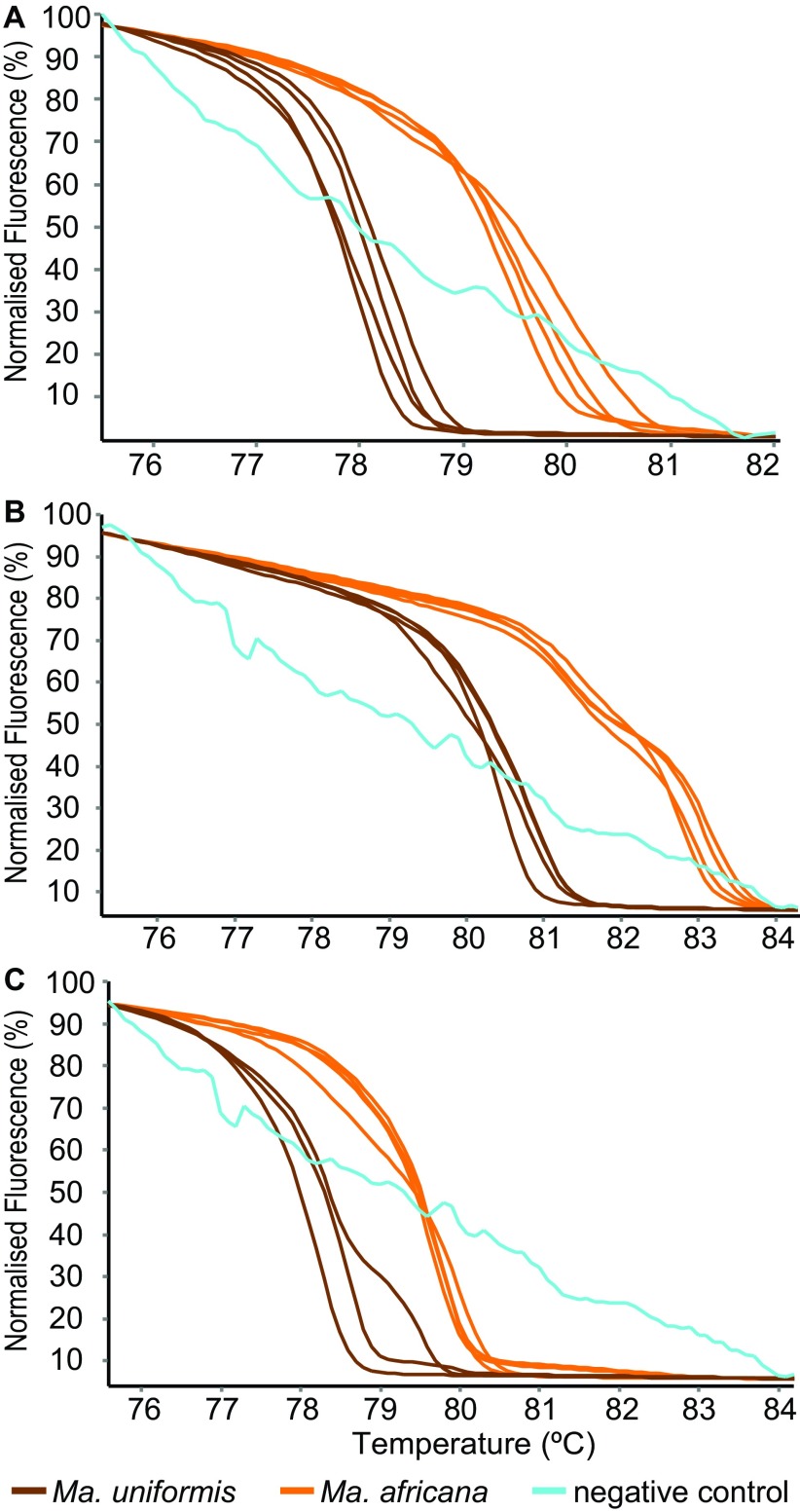
HRM profiles of two *Mansonia* species. *Mansonia uniformis* and *Ma*. *africana* mosquitoes were differentiated by PCR-HRM using the ( **A**) COI-AnophF/HCO2198R, ( **B**) MOS-CO1 and ( **C**) CYT B primer pairs.

**Figure 2.**
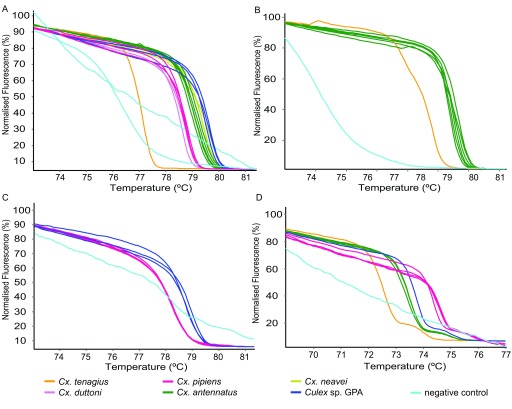
HRM profiles of *Culex* species. *Culex* species were differentiated by PCR-HRM using the ( **A**) COI-AnophF/HCO2198R, ( **B**) CYT B, ( **C**) Uni-Minibar-JV, and ( **D**) Mos-ND1 primer pairs.

**Figure 3.**
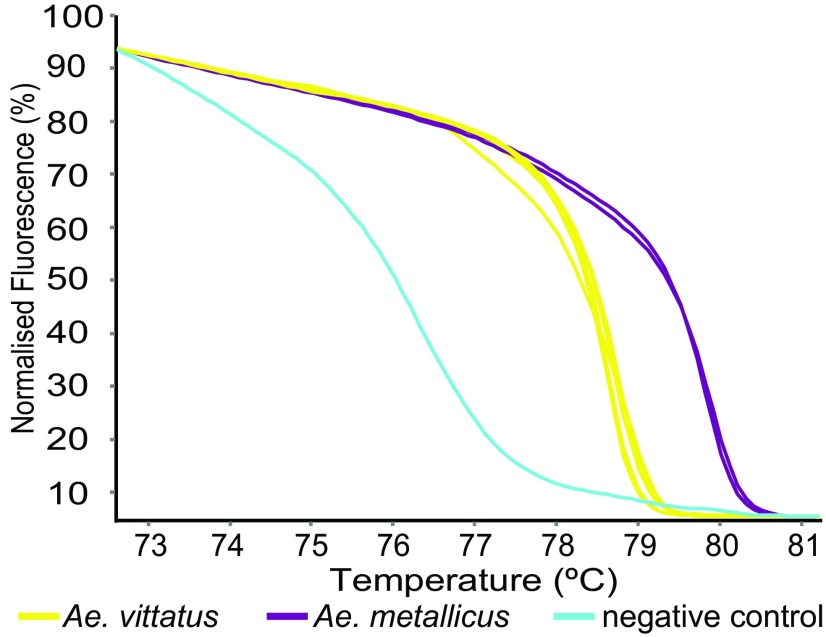
HRM profiles of *Aedes* mosquitoes. *Aedes vittatus* and *Ae. metallicus* were differentiated by PCR-HRM using the COI-AnophF/HCO2198R primer pair.

**Figure 4.**
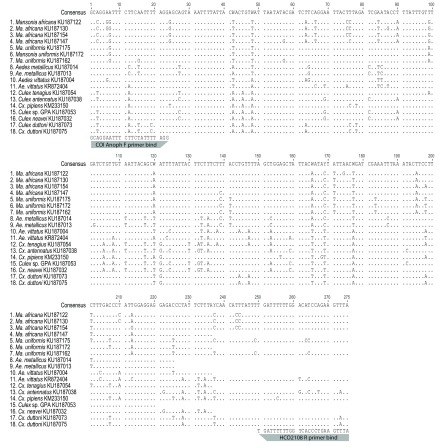
Single nucleotide polymorphisms (SNP) between mosquito species separated by the COI-AnophF/HCO2108R primer pair amplicons. Polymorphic sites vary more between than within species.

We differentiated 12 mosquito species in the *Aedes* (two) *, Anopheles* (two) *, Culex* (six), and *Mansonia* (two) genera by HRM analyses ( Table 4). The *COI* sequences of some of the mosquito samples analyzed and differentiated were obtained during a previously published mosquito diversity study ^6^ and their respective GenBank Accession numbers are listed in Table 1 and Table 2. Despite the fact that the COI-AnophF/HCO2198R primers were originally designed based on *Anopheles* mitochondria genome alignments, they were most efficient in differentiating among *Mansonia* ( *Ma. africana* and *Ma. uniformis* ( Figure 1A)), *Culex* ( *Cx. neavei* and *Cx. duttoni*, *Cx. tenagius* and *Cx. antennatus*, and two genetic variants of *Cx. pipiens* ( Figure 2A)), and *Aedes* ( *Ae. vittatus* and *Ae. metallicus* ( Figure 3)) mosquitoes ( Table 4). Indeed, the DNA sequences flanked by the COI-AnophF/HCO2198R primers included multiple polymorphic sites in species within these genera ( Figure 4). Although there are SNPs within species DNA that resulted to the slight changes observed in their HRM profiles, the SNPs across species were enough to distinguish between them.

**Table 4.** Differentiation of mosquito species using the six primer pairs amplifying four loci.

Mosquito genera	*COI*			*cyt b*	ND1	IGS
	COI-AnophF/HCO2198R	Mos-CO1-JV	Uni-Minibar-JV	CYT B	Mos-ND1	AgamUni
*Anopheles*	DNS	DNS	DNS	DNS	DNS	Separated *An. gambiae* from *An. arabiensis*
*Mansonia*	Separated *Ma. africana* from *Ma. uniformis*	Separated *Ma. africana* from *Ma. uniformis*	DNS	Separated *Ma. africana* from *Ma. uniformis*	DNS	DNS
*Aedes*	Separated *Ae. vittatus* from *Ae. metallicus*	DNS	DNS	DNS	DNS	DNS
*Culex*	Separated *Cx. tenagius* from *Cx. antennatus*, Separated *Cx. pipiens* from *Culex* sp. GPA, Separated *Cx. neavei* from *Cx. duttoni*	DNS	Separated *Cx. pipiens* from *Culex* sp. GPA	Separated *Cx. tenagius* from *Cx. antennatus*	Separated *Cx. tenagius* from *Cx. antennatus*, Separated *Cx. pipiens* from *Culex* sp. GPA	DNS
*Aedeomyia*	DNS	DNS	DNS	DNS	DNS	DNS
*Mimomyia*	DNS	DNS	DNS	DNS	DNS	DNS
*Coquillettidia*	DNS	DNS	DNS	DNS	DNS	DNS

DNS means did not separate. *COI* means cytochrome c oxidase subunit 1. *cyt b* means cytochrome B. ND1 means NADH dehydrogenase subunit 1. IGS means intergenic spacer region.

**Figure 5.**
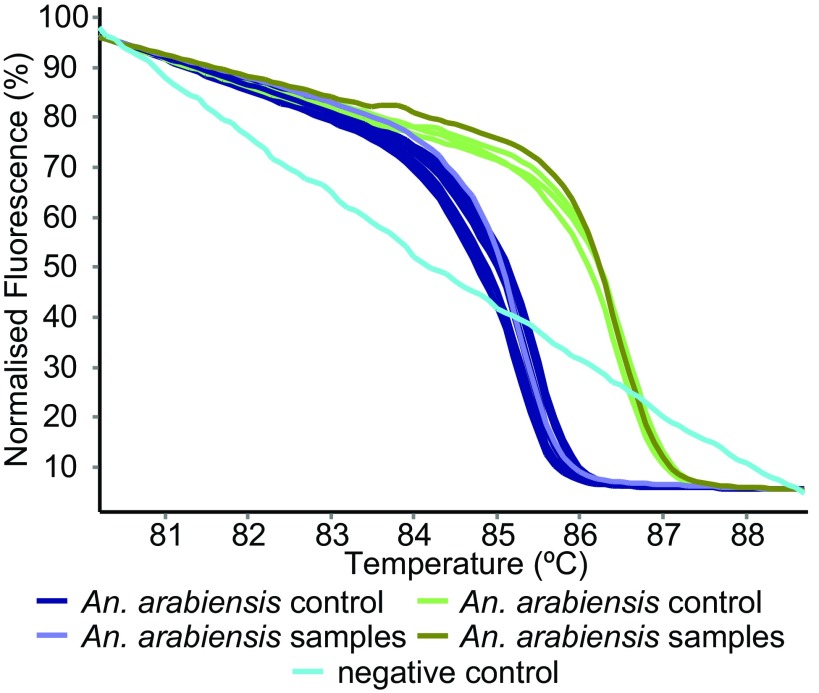
HRM profiles of *Anopheles* mosquitoes. Two sibling species of *Anopheles gambiae s.l.* were differentiated by PCR-HRM using the AgamUni primer pair.

*Mansonia africana* and *Ma. uniformis* could also be differentiated by Mos-COI-JV ( Figure 1B) and CYT B ( Figure 1C) PCR-HRM analysis. Some *Culex* species were similarly differentiated by HRM based on their CYT B, Uni-Minibar-JV and Mos-ND1 ( Figure 2B–D) primer pair PCR products. The morphologically indistinguishable *Cx. tenagius* and *Cx. antennatus* were distinguished only by the COI-AnophF/HCO2198R, CYT B and ND1 primers ( Figure 2A, B and D). Similarly, HRM analysis of only two of the *COI* (COI-AnophF/HCO2198R and Uni-Minibar JV) and the ND1 primer pairs grouped morphologically identical and difficult to differentiate *Cx. pipiens* into two distinct clades: one with *Cx. pipiens* voucher sequences from GenBank (KF919189) and those with a sequence that we identified as *Culex* sp. GPA ^6^ (GenBank accessions KU380352, KU380455, KU380394) ( Figure 2A, C and D; Table 4). However, unlike the *COI* HRM profiles ( Figure 2A, B), the ND1 HRM profiles ( Figure 2D) of *Cx. pipiens* amplicons showed a melting temperature shift of to the right (higher temperature) compared to the *Culex* sp. GPA amplicons, possibly due to greater GC richness of *Cx. pipiens* at this locus ^28^. Similarly, the IGS primers (AgamUni) differentiated *Anopheles gambiae s.s.* from *An. arabiensis* ( Figure 5). In addition, the COI-AnophF/HCO2198R primers were also used to separate *Cx. neavei* from *Cx. duttoni* ( Figure 2A), which belong to the same subgenus of *Culex* mosquitoes.

HRM analysis of all the six primer pairs could not differentiate *Aedeomyia* ( *Ad. africana* and *Ad. furfurea*), *Mimomyia* ( *Mi. hispida* and *Mi. splendens*) and *Coquillettidia* ( *Cq. aurites*, *Cq. chrysosoma*, *Cq. fuscopennata*, *Cq. metallica*, *Cq. microannulatus*, *Cq. pseudoconopas* and *Cq. versicolor*) species ( Table 4) or among *An. funestus* and *An. coustani* species complexes.

## Discussion

We compared six pairs of primers for their potential to differentiate at least two morphologically similar mosquito species within each of seven mosquito genera by PCR-HRM analysis and identified suitable markers for differentiating species within *Anopheles*, *Aedes, Culex* and *Mansonia* mosquitoes. However, none of the markers were suitable for HRM analysis to distinguish among species of *Aedeomyia*, *Mimomyia* or *Coquillettidia* genera mosquitoes. Also, *Cx. watti*, which can be misidentified morphologically as *Cx. duttoni* or *Cx. pipiens*, could not be differentiated by PCR-HRM analyses. Nonetheless, we were able to distinguish *Ma. africana* from *Ma. uniformis, An. gambiae s.s.* from *An. arabiensis* (sibling species of *An. gambiae s.l.*), *Ae. vittatus* from *Ae. metallicus*, as well as *Cx. neavei* from *Cx. duttoni*, *Cx. tenagius* from *Cx. antennatus* and two cryptic sympatric species of morphologically identical *Cx. pipiens*. Most notably, the two *Cx. pipiens* species with distinct COI barcode sequences ^6^ were indeed first identified by HRM analysis of numerous samples ^6^. Thus, the relative economy of HRM analysis compared to sequencing facilitates the rapid identification of cryptic species.

Surprisingly, HRM analysis of PCR products from the COI-AnophF/HCO2198R primers, which were designed for *Anopheles*, could not distinguish between these sibling species, yet were most effective in discriminating species within the *Mansonia*, *Aedes* and *Culex* genera, including between the cryptic *Culex pipiens* species. *Anopheles gambiae* and *An. arabiensis* were only distinguished using the IGS gene, which was also designed for *An. gambiae* ^2^ and is routinely used for distinguishing these sibling species by conventional PCR ^29^ and HRM analysis ^14^. In contrast, species complexes of *An. coustani* and *An. funestus* were not differentiated with any of the primers. The data suggest that COI ^30^, *cyt b* and ND1 loci may be unsuitable for distinguishing among *Anopheles* sibling species. Similarly, the *Aedes* species could only be differentiated by the COI-AnophF/HCO2198R primers. This could be as a result of more recent speciation, insufficient to allow for sibling species resolution at these markers. Such scenarios have been observed for recent or rapidly evolving groups, such as the Cichlid fishes of eastern Africa, where mitochondrial divergence is not concordant with morphological variations ^31^.

In contrast, *Ma. africana* and *Ma. uniformis* were separated by the COI and *cyt b* loci, but not by the ND1 and IGS gene primers and *Culex* species were variably differentiable by all markers, except IGS. For both *Mansonia* and *Culex*, as with *Aedes*, the COI-AnophF/HCO2198R primers were most sensitive in discriminating morphologically indistinct species. This highlights the power of the COI barcode region for identifying diverse cryptic species ^32^. The SNPs present in the *COI* genes of the ten mosquito species confirms that the *COI* gene is conserved and polymorphic enough to differentiate these species even in cases of morphological misidentification. The polymorphisms between species were enough to robustly separate them based on their HRM profiles, while sequence polymorphisms within species were too few to significantly alter their HRM profiles.

We, therefore, recommend the initial use of the COI-AnophF/HCO2198R primers Bar-HRM to differentiate *Mansonia*, *Culex* and *Aedes* mosquito species and the IGS primers for anopheline mosquito identification ^2, 14, 33^ by HRM. The inability of all the six primer pairs to differentiate many mosquito species among all seven genera tested is an indication that the genetic diversity of many mosquito species is complicated and still poorly understood. Also, the number (sample size) of many of the analyzed mosquito species was small (<3) because these species were scarcely present in the study areas. More samples (≥3) should be used and more study areas should be sampled in subsequent studies to test genetic differentiation of mosquito species ^34^. Additional polymorphic DNA loci should also be identified, tested and used in combination with existing ones for the identification of mosquito species, especially among species complexes and across genera.

## Conclusions

This study shows that specific PCR markers can be used to distinguish closely related species of mosquitoes using HRM analysis. We distinguished two sibling species of *An. gambiae s.l.*, two species each of *Mansonia* and *Aedes*, and six species, including cryptic species, of *Culex* using six pairs of primers targeting the mitochondrial and ribosomal genes. HRM is a low cost (<$1 per reaction), effective tool that enhances culicine and anopheline mosquito identification and may also reveal population differences in conserved mitochondrial sequences. This approach can improve vector surveillance associated with *Plasmodium* (malaria) or arbovirus transmission and inform targeted vector control strategies.

## Data availability

The data referenced by this article are under copyright with the following copyright statement: Copyright: © 2016 Ajamma YU et al.

Data associated with the article are available under the terms of the Creative Commons Zero "No rights reserved" data waiver (CC0 1.0 Public domain dedication). 

All sequence data associated with this manuscript are freely available in GenBank. All relevant accession numbers are listed in Table 1 and Table 2.

*F1000Research*: Dataset 1. Raw Rotor-Gene Q HRM data files (.rex), viewable using Rotor-Gene Q software (Qiagen), 10.5256/f1000research.9224.d130565 ^36^
