# Supplementary material for: Rapid and high throughput molecular identification of diverse mosquito species by high resolution melting analysis
Source: F1000Res. 2016 Aug 11;5:1949. [Version 1] doi: 10.12688/f1000research.9224.1 (PMC5031131; doi:10.12688/f1000research.9224.1)
Supplement: Peer review history

# Peer review: Referee response for version 1

**DOI:** 10.5256/f1000research.9928.r16385

**Published:** 2016-09-19

**Authors:** Hwa Chia Chai

**Affiliations:** 1 Department of Biomedical Science, Faculty of Medicine, University of Malaya, Kuala Lumpur, Malaysia

Overall, this is a well-written article on the development of HRM for identification of different species of mosquitoes. The authors also mentioned about the limitations of the study. However, the six pairs of primers used in this study did not seem to have clear directions or purpose. Did the authors want to come out with a pair of universal primers to differentiate all the clinically important mosquito species mentioned in the article? Or one specific primer pair for each genus of mosquitoes? Or there is other intention? Although the authors showed the ability of primer pairs to discern some mosquito species, for instance, COI-AnophF/HCO2198R could distinguish *Mansonia* spp., *Aedes* spp. and *Culex* spp., how about the species of the same genera that could not be discriminated? It would be more focused and directional if the primer pairs are genus-specific.

 Methods:

1. It would be great if the clinical importance of or disease transmitted by each mosquito genus/species is listed in Table 1 and Table 2 so that the readers understand the significance of discriminating the mosquito species.
2. Please mention the T _m_ for each primer in Table 3.
3. Please elaborate more on the primer design: on what basis the authors design the primers and for what reason they want to amplify those regions with amplicon sizes mentioned in Table 3.
4. What was the amount of DNA template used for HRM analyses?

 

 Results:

1. Please show the limitation of detection for each assay in the detection of each mosquito species.
2. Was auto-calling mode used for clustering? What was the confidence interval? Please mention the mean T _m_ for each species with standard deviation.
3. Figure 2A, B, D: There was only one sample of *C. tenagius* included in the analysis. It is difficult to conclude the melting profile of this species if there was only one sample available.
4. In Table 1, COI-AnophF/HCO2198R has been shown to be able to separate *Cx. tenagius* from *Cx. antennatus*, *Cx. pipiens* from *Culex sp. GPA*, and *Cx. neavei* from *Cx. duttoni*. Since I am not an expert in mosquitoes, is there any specific reason the authors wanted to report the separation between two species of Culex rather than reporting it as a separation between all species?   
5. In Figure 2A, the melt curves of *Cx. antennatus*, *Cx. neavei*, *Culex *sp. GPA and *Cx. pipiens* looks closely apart and hard to differentiate. Was the clustering auto- or manually called? What was the confidence interval if it was auto-called? What is the possibility of all these species being present or analysed at the same time? It would be hard to differentiate them if all of them are present in a same run of HRM analysis.
6. How do the melt curves of the unseparated species appear as compared to those in the same genus which could be differentiated? For instance, *Cx. perexiguus* vs. *Cx. tenagius*, *Cx. antennatus*, *Cx. pipiens*, *Culex sp. GPA*, *Cx. neavei* and *Cx. duttoni*. And also melting curves of *Aedeomyia* ( *Ad. africana* and *Ad. furfurea*), *Mimomyia* ( *Mi. hispida* and *Mi. splendens*) and *Coquillettidia *( *Cq. aurites*, *Cq. chrysosoma*, *Cq. fuscopennata*, *Cq. metallica*, *Cq. microannulatus*, *Cq. pseudoconopas* and *Cq. versicolor*) species (Table 4) or among *An. funestus* and *An. coustani* species complexes, which could not be differentiated in this study.
7. In Figure 4, intraspecies variation in the target sequence is seen, for instance, in the four strains of *Ma. africana*. Although the authors did mention on Page 6 “Although there are SNPs within species DNA that resulted to the slight changes observed in their HRM profiles, the SNPs across species were enough to distinguish between them”. I am not sure how confident it is to employ these assays in the presence of intraspecies variations, plus the sample size may be too small to validate the assays. 
8. Figure 5: please revise the figure legend.

 

 Discussion:

1. On page 8, 2 ^nd^ paragraph, the authors mentioned “Surprisingly, HRM analysis of PCR products from the COIAnophF/HCO2198R primers, which were designed for Anopheles, could not distinguish between these sibling species….” Can the authors explain the possible reason for this?
2. Again, since I am not a mosquito expert, are the mosquitoes of the same genus morphologically identical? If the assays are successfully developed, are the mosquitoes going to be morphologically identified prior to subjecting them to HRM analysis? How are the authors going to decide which primer pairs to use later in the mosquito identification?

I have read this submission. I believe that I have an appropriate level of expertise to confirm that it is of an acceptable scientific standard.

# Peer review: Referee response for version 1

**DOI:** 10.5256/f1000research.9928.r15904

**Published:** 2016-09-12

**Authors:** Michael Zianni

**Affiliations:** 1 Thermo Fisher Scientific, Carlsbad, CA, USA

Ajamma have an article on the use of the technique high resolution melt analysis towards the identification of morphologically indistinct species of mosquito. The specific goal is to expand the current set of primers in the research literature in order to identify more species from multiple genera of mosquitoes.

 The methodology is clear with sufficient details for it to be reproduced by listing all appropriate reagents, DNA primer sequences and real-time PCR instrumentation. I do recommend adding the criteria by which the melt curves were deemed to be sufficiently different to allow identification of the species as compared to the "Did Not Separate" state as reported in Table 4.

 Figures 1 - 3 and 5 are clear and support the results summarized in table 4. I appreciate the authors efforts to repeat and report data from the previously published primers ("AgamUni") as a point of comparison. Appropriate controls were used with (1) water as a negative control for amplification and (2) samples from defined colonies and samples previously sequenced as positive controls. The most significant limitation is the number of replicates, and the diversity of sample collection points for each species.   The authors clearly acknowledge these limitations in the conclusion and clearly state the need for additional samples to asses the intra-specific variation which is critically important information to make this method highly useful.

 In summary, the paper is clearly and concisely written with 1 minor recommendations for additional information on the method.  The goals of the research are clearly stated, and the results as well as the conclusions support the goals.  The researchers have achieved the goals by identifying and confirming at least one primer pair for each of 4 genera that identify various species that are difficult to identify by morphology alone.

I have read this submission. I believe that I have an appropriate level of expertise to confirm that it is of an acceptable scientific standard.
